# Applying systems thinking to task shifting for mental health using lay providers: a review of the evidence

**DOI:** 10.1017/gmh.2017.15

**Published:** 2017-07-31

**Authors:** D. Javadi, I. Feldhaus, A. Mancuso, A. Ghaffar

**Affiliations:** 1 Alliance for Health Policy and Systems Research, WHO, Geneva, Switzerland; 2 Harvard T.H. Chan School of Public Health, Boston, Massachusetts, USA; 3 Johns Hopkins Bloomberg School of Public Health, Baltimore, Maryland, USA

**Keywords:** Community, health system, mental health, systems thinking, task-shifting

## Abstract

**Objective.:**

This paper seeks to review the available evidence to determine whether a systems approach is employed in the implementation and evaluation of task shifting for mental health using lay providers in low- and middle-income countries, and to highlight system-wide effects of task-shifting strategies in order to better inform efforts to strength community mental health systems.

**Methods.:**

Pubmed, CINAHL, and Cochrane Library databases were searched. Articles were screened by two independent reviewers with a third reviewer resolving discrepancies. Two stages of screens were done to ensure sensitivity. Studies were analysed using the World Health Organization's building blocks framework with the addition of a community building block, and systems thinking characteristics to determine the extent to which system-wide effects had been considered.

**Results.:**

Thirty studies were included. Almost all studies displayed positive findings on mental health using task shifting. One study showed no effect. No studies explicitly employed systems thinking tools, but some demonstrated systems thinking characteristics, such as exploring various stakeholder perspectives, capturing unintended consequences, and looking across sectors for system-wide impact. Twenty-five of the 30 studies captured elements other than the most directly relevant building blocks of service delivery and health workforce.

**Conclusions.:**

There is a lack of systematic approaches to exploring complexity in the evaluation of task-shifting interventions. Systems thinking tools should support evidence-informed decision making for a more complete understanding of community-based systems strengthening interventions for mental health.

## Introduction

Globally, mental health accounts for a large and growing burden of disease (Whiteford *et al.* 2013). Recent estimates from the WHO Mental Health Surveys indicate an interquartile range of lifetime DSM-IV disorder prevalence between 18.1% and 36.1% (Kessler *et al.* 2009). According to the Global Burden of Disease Study, between 2005 and 2013, disability-adjusted life-years attributed to mental and neurological disorders increased by 9.7% and 16.1%, respectively (Murray *et al.* 2015*a*). Despite this burden, a study across 17 countries demonstrated that only 20% of persons with common mental disorders (CMDs) received treatment in the year prior to the survey, with only 10% receiving minimally adequate treatment (Wang *et al.* 2007). Availability and scale-up of essential health services to achieve health system goals is often impeded by health workforce shortages (WHO, 2006). This is especially true of mental health in low- and middle-income countries (LMICs), where availability of services is not matched to population needs (Weinmann & Koesters, 2016). The World Health Organization (WHO) estimates that there is a need for 1.18 million mental health workers to move towards closing the mental health treatment gap (Fulton *et al.* 2011).

In LMICs, not only are there limited mental health services available, utilization of existing services is also poor for a multitude of reasons, including geographic, cultural, and financial access barriers (Murthy, 2011; van Ginneken *et al.* 2013; Chowdhary *et al.* 2014; Joshi *et al.* 2014; Chibanda *et al.* 2015; Weinmann & Koesters, 2016). Mental health service delivery is highly context-specific with culturally defined interpretations of stigma, trust, and utility affecting success and impact (Murthy, 2011; van Ginneken *et al.* 2013; Weinmann & Koesters, 2016). Integration of mental health services into primary care presents a strategic opportunity to overcome some of these access barriers and reach the largest number of people while minimizing stigma and discrimination (WONCA, 2008). Integration of mental health services is also in line with the essential public health function of early diagnosis and prevention; it requires primary care providers to be trained in identifying poor mental health and taking action towards treatment (WONCA, 2008). Mental and physical health are interconnected, and early detection can lead to improved health outcomes and increase cost-effectiveness for the health system (WONCA, 2008; Levin & Chisholm, 2015). However, effective integration requires strengthened primary care systems. The value of lay provider programmes in strengthening these systems towards universal health coverage, which includes provisions for mental health, has been recognized globally (Schneider & Lehmann, 2016). For the purposes of this paper, lay providers are defined broadly as individuals who may or may not have basic literacy skills or some form of formal post-secondary education with subsequent informal or formal pre-service training (Olaniran *et al*. 2017). They are often unpaid or may receive an allowance based on the programme (Olaniran *et al*. 2017).

### Recognizing task shifting as a system intervention for mental health

In resource-poor settings, task shifting has been an effective approach to addressing health workforce challenges and strengthening systems for mental health (Eaton *et al.* 2011; Kakuma *et al.* 2011). Several systematic reviews have supported the use of task shifting for mental health focused on specific populations, such as with people living with HIV/AIDS or mothers with postpartum depression (Rahman *et al.* 2013; van Ginneken *et al.* 2013; Chowdhary *et al.* 2014; Atif *et al.* 2015; Chibanda *et al.* 2015). Task shifting includes shifting service delivery of specific tasks from professionals with higher qualifications to those with fewer qualifications or creating a new cadre with specific training (WHO, 2007*b*). It is meant to alleviate the heavy workload of specialists and to ensure that those with no access to specialists have a means of accessing some level of mental health services (Patel *et al.* 2007). By shifting service delivery for less complex cases to lay providers, the system allows mental health specialists to focus on more complex cases with the hope that quality of care delivery will also improve (Weinmann & Koesters, 2016).

Task shifting requires various parts of the system to be working in harmony in order to be successful (GHWA, 2007; Yaya Bocoum *et al.* 2013). Conditions such as regular supervision, availability of resources and tools, access to medicines, quality training, and exposure to technological updates through in-service training are enabling factors in ensuring successful redistribution of tasks among health workforce teams (Yaya Bocoum *et al.* 2013; Agyapong *et al.* 2016). Buy-in and acceptance of task-shifting interventions across a wide range of stakeholders is also important in their success (Yaya Bocoum *et al.* 2013). For example, perceptions of a loss of hierarchal structures, shift in earnings, and burden of supervision are examples of barriers that higher professional cadres may experience regarding task shifting (Niekerk, 2008; Philips *et al.* 2008; Zachariah *et al.* 2009). Therefore, task shifting is a system-wide intervention that has implications beyond the players and programmes directly involved in its implementation; it reallocates resources across the health system to trigger change.

### Using systems thinking to evaluate the impact of task shifting

With the recognition that task shifting for mental health is a system-wide intervention, understanding its potentially far-reaching implications and impact across the system becomes valuable for appropriate decision making, health system planning, and implementation of interventions. System-wide effects can be captured using the suite of tools available in systems thinking to collect information across a multitude of stakeholders and mechanisms involved in a given context (AHPSR, 2009; Peters, 2014). The six building blocks of the health system – service delivery, health workforce, information technology, medical products, financing, and leadership – are made dynamic, adaptive, and interactive through a systems thinking lens as it is designed to explore how different elements are connected in a system and the impact and implications of these connections (Table 1) (Adam *et al.* 2012). Systems thinking also incorporates another key health systems element: communities and people (Adam *et al.* 2012). Therefore, in our application of the building blocks approach, we have added a seventh to account for communities and people. By enhancing understanding of different perspectives across multiple interacting agents, the changing context in which they interact, and the changes resulting from new patterns created over time, systems thinking can serve as an important policy toolkit (Adam *et al.* 2012; Peters, 2014).

**Table 1.** Building blocks of the health system (WHO, 2010)

Building block	Defining characteristics	Source of data
Service delivery	Considers comprehensiveness of services provided, accessibility, coverage, continuity of high-quality, person-centred care across network of services, and efficient and accountable management of these services	Routine health facility monitoring systems Health facility surveys and assessments
Health workforce	Encompasses ‘workers in different domains of health systems, such as curative, preventive and rehabilitative care services as well as health education, promotion and research’. (WHO, 2010) Related data needed for effective management includes comprehensive, reliable, timely information on numbers, demographics, skills, services being offered, and factors influencing recruitment and retention (payment structures, treatment, supervision, training, work burden, work environment, skill-mixing, etc.) of human resources for health	Population census Labour force survey Health facility assessment Civil services payroll registry Registries of professional regulatory bodies
Health information systems	Functional health information systems exist where countries have: health survey plans that cover all priority health topics, two or more data points available for maternal mortality, child mortality, coverage, smoking and nutrition; birth and death registrations; ICD-10 used in district hospitals to report on deaths; census completed; HIV prevalence; health facility data; data quality assessment reports; health statistics web site; national health accounts exercise; health systems performance assessment; and institutional mechanisms for analysis of health data.	Health surveys Birth and death registration Census Health facility reporting Health system resource tracking
Medicines and medical devices	Considers ‘equitable access to essential medical products, vaccines and technologies of assured quality, safety, efficacy and cost effectiveness, and their scientifically sound and cost-effective use’. (WHO, 2010) Aspects that impact access include: national policies and guidelines, price negotiation, reliable manufacturing practices, effective procurement, supply and storage, distribution systems, leakage protection, support for rational use, and awareness-raising for both providers and patients	Facility surveys Essential medicines lists Key informant interviews and surveys Legislative review Household surveys
Financing	‘Concerned with the mobilization, accumulation and allocation of money to cover the health needs of the people, individually and collectively, in the health system… the purpose of health financing is to make funding available, as well as to set the right financial incentives to providers, to ensure that all individuals have access to effective public health and personal health care’ (WHO, 2010)	National health accounts Household expenditure surveys Health insurance enrolment records Government expenditure accounts
Leadership and governance	Considers ‘strategic policy frameworks […] combined with effective oversight, coalition-building, regulation, attention to system design and accountability’. (WHO, 2010)	National health policy reviews Rule-based indicators: existence of specific policies Outcome-based indicators: span across other building blocks for good performance overall
Community and people	Considers community voice, engagement and consultation. Includes context-specific considerations based on the community's needs	Community meetings; programme implementation documents

### Applying evidence for success in capacity building for mental health care

The growing burden of disease attributed to mental health calls for approaches that strengthen the capacity of the health system to equitably and appropriately address the wide range of mental and neurological disorders (Whiteford *et al.* 2013). The mhGAP (Global Mental Health Gap Action Programme) was launched in 2008 to provide technical guidance, tools and training to help address the challenges of availability in resource-poor settings (WHO, 2008). Global mental health has seen attention in academic circles through special series in *The Lancet* and *PLoS*, which highlight integration of mental health into primary care as a key strategy (Patel *et al.* 2007; Patel & Thornicroft, 2009).

Integration of mental health into health systems, especially in primary care systems, is not without its challenges, particularly in resource-poor settings (Patel *et al.* 2010*a*; Eaton *et al.* 2011; Weinmann & Koesters, 2016). Poor policy implementation, inadequate human resource allocation to support the process, poor community engagement, and low access to medicines are among the challenges of integration (Patel *et al.* 2007; Eaton *et al.* 2011; van Ginneken *et al.* 2013). Systems thinking contributes to documenting the system-wide impact of a given intervention, as well as enhancing the ability to predict both intended and unintended consequences of the intervention, critical in designing successful large-scale reform.

Few studies focus on the wider impact of task shifting across the health system and, likewise, the scale-up of mental health strategies (Eaton *et al.* 2011; Yaya Bocoum *et al.* 2013). This weakness in the literature undermines the complexity of interactions and changes that take place in health systems, and stymies the potential scale-up and sustainable integration of promising task-shifting strategies (Adam *et al.* 2012; Yaya Bocoum *et al.* 2013). To ensure that LMICs can expand large-scale mental health strategies and achieve integration into primary care, a system-wide approach can be an effective tool in understanding, evaluating, and implementing bespoke strategies (WHO, 2007*b*; AHPSR, 2009). This paper reviews the available evidence to determine whether a systems approach is employed in the implementation and evaluation of task shifting for mental health using lay providers in LMICs. It seeks to highlight system-wide effects of task-shifting strategies in order to better inform efforts to strengthen community mental health systems.

## Methods

### Search strategy

The electronic databases of PubMed, CINAHL, and Cochrane were searched between 5 September 2016 and 30 October 2016 (Annexure 1). The search strategy consisted of three concepts: (1) lay providers, including community health workers, health aides, local references to community health workers such as accredited social health activists, non-physician health workers, community-based practitioners, and other associated terms; (2) mental health, including the standard set of disorders under the definition of CMDs such as anxiety, depression, dementia, schizophrenia, and substance abuse, as well as strategies for treatment such as supportive counselling, cognitive behavioural therapy, and others; and (3) LMIC setting, as this study is focused on alternatives for delivery of mental health services in resource-poor settings. These concepts were expanded to include similar terms and combined using ‘and’ to build the search. Further, the references of included articles were searched to identify additional citations that were not captured in the search as a means of ensuring the robustness of the study. These were included when the full text satisfied the inclusion criteria of being set in an LMIC, focusing on mental health and evaluating a task-shifting strategy of service delivery from providers with higher or more specialized qualifications to those with lower qualification. However, all eligible references were already captured in the search. Search was limited to publications between January 1996 and September 2016.

After completing the electronic search, the titles and abstracts of all identified articles were independently reviewed by two authors, who assessed whether the article should be included or excluded according to pre-defined criteria. These criteria are included in Table 2. Articles that met any of the criteria for exclusion were eliminated. In the first round of screening, articles meeting at least four of five criteria for inclusion based on titles and abstract review, were included. In the case of inter-rater disagreement, a third reviewer was consulted on the inclusion or exclusion of the article in question. The third reviewer was blinded and has expertise in health systems research. Articles intended for inclusion were combined in a Microsoft Excel spreadsheet and any duplicates were removed. Full-text versions of identified articles were examined in order to reassess inclusion based on articles meeting all five criteria before establishing the final set to be included in the study. A two-stage approach to inclusion was employed to ensure sensitivity.

**Table 2.** Inclusion and exclusion criteria

Inclusion	Exclusion
The research article evaluated an intervention/implementation strategy Mental health was a significant component of the intervention/implementation strategy The intervention/implementation strategy was introduced in a LMIC The intervention/implementation strategy involved task-shifting to lay providers Lay providers had fewer than 3 years of training	The peer-reviewed publication was not a research article The peer-reviewed publication was not in English Pilot study

For inclusion in this review, the study must have: (1) evaluated the implementation and/or impact of an intervention; (2) had significant focus on mental health; (3) been set in an LMIC; (4) employed task-shifting strategies where service delivery was transformed from a professional cadre with higher qualification to lay providers with lower qualifications and minimal mental health training; and (5) involved training of lay providers was limited to fewer than 3 years. The training criteria was articulated with input from a health workforce specialist in order to keep the focus on task shifting to providers with fewer qualifications without excluding task shifting to qualified providers who lack specialized mental health training as we considered this relevant to our study. Where length of training was not specified, we used our collective judgment to determine whether task shifting was towards a provider with minimal mental health training. An expanded interpretation of evaluation was used to include both quantitative and qualitative studies that reported on randomized control trials, cohort studies with before and after measures, survey and/or observational assessments of stakeholder perceptions, acceptability and satisfaction, case studies, and analysis of qualitative data.

### Data extraction and analysis

Two study authors read all included full texts and extracted the following data: setting, year of publication, aim of study, type of intervention, sample size, outcomes measured, results, health system implication(s), and barriers and facilitators of implementation. Critical Appraisal Skills Programme (CASP) tools were used to assess the quality of the studies (CASP, 2016). The initial screening questions (see Table 3) were used to ensure that included studies met minimal quality standards. Risk of bias and limitations of included studies were then assessed using more detailed items found on CASP checklists for different types of studies (see Table 4).

**Table 3.** CASP screening questions

Screening question	Considerations
Was there a clear research question/objective?	What was the goal of the research? Why was it thought important? Is the question focused in terms of the population, risk factors, and/or outcomes studied?
Was the methodology/research design used appropriate to address the aims of the research?	Do authors provide justification for the research design? Are there selection bias and/or generalizability issues? Were effects of the intervention identified, measured, and valued appropriately?

**Table 4.** CASP quality checklist

Type of study design	Detailed questions
Randomized controlled trial	Was the assignment of patients to treatment randomized? Were all of the patients who entered the trial properly accounted for at its conclusion? Were patients, health workers and study personnel ‘blind’ to treatment? Were the groups similar at the start of the trial? Aside from the experimental intervention, were the groups treated equally?
Case control study	Were the cases recruited in an acceptable way? Were the controls selected in an acceptable way? Was the intervention / exposure accurately measured to minimize bias? Have the authors considered all potential confounding factors in the design and/or analysis?
Cohort study	Was the cohort recruited in an acceptable way? Was the intervention / exposure accurately measured to minimize bias? Was the outcome accurately measured to minimize bias? Have the authors considered all important confounders? Was the follow up of subjects complete and/or long enough?
Qualitative study	Was the recruitment strategy appropriate to the aims of the research? Was the data collected in a way that addresses the research issue? Has the relationship between researcher and participants been adequately considered? Have ethical issues been taken into consideration? Was the data analysis sufficiently rigorous? Is there a clear statement of findings?
Economic evaluation	Was a comprehensive description of the competing alternatives given? Does the paper provide evidence that the programme would be effective? Were all effects of the intervention identified, measured and valued appropriately? Were all important and relevant resources required and health outcome costs for each alternative identified, measured in appropriate units and valued credibly? Were costs and consequences adjusted for different times at which they occurred (discounting)? Was an incremental analysis of the consequences and cost of alternatives performed? Was an adequate sensitivity analysis performed?

To determine whether a system-wide approach was taken in the evaluation of the intervention and to identify system-wide effects when available, authors identified features of interventions relevant to the WHO building blocks framework as well as systems thinking characteristics used in the study (AHPSR, 2009). Systems thinking characteristics considered included: capturing perceptions and interactions of multiple interacting agents, network analysis, mapping of contextual factors, process mapping, describing feedback mechanisms, and other approaches that could inform system dynamics modelling (Peters, 2014). Manuscripts were coded for identification of barriers, facilitators, and outcomes that were relevant to each of the six building blocks: (1) Service Delivery, (2) Health Workforce, (3) Information Technology, (4) Medicines & Medical Devices, (5) Financing, and (6) Leadership and Governance (WHO, 2007*a*). A seventh building block for communities and people was also included in data abstraction. Authors also made note of the range of stakeholders consulted in the study. The building blocks model allowed for a systematic way to determine whether the impact of the intervention was assessed beyond the specific building blocks in which they were implemented (i.e. health workforce and service delivery in the case of task shifting for mental health). By looking at the level and range of stakeholder engagement, we were able to identify instances were roles and interactions of stakeholders not directly involved in the intervention were explored, as is customary in systems thinking. Use of system dynamics theory, causal loop diagrams, and other system modelling techniques were also included in the extraction criteria, but none were found.

## Results

**Fig. 1.**
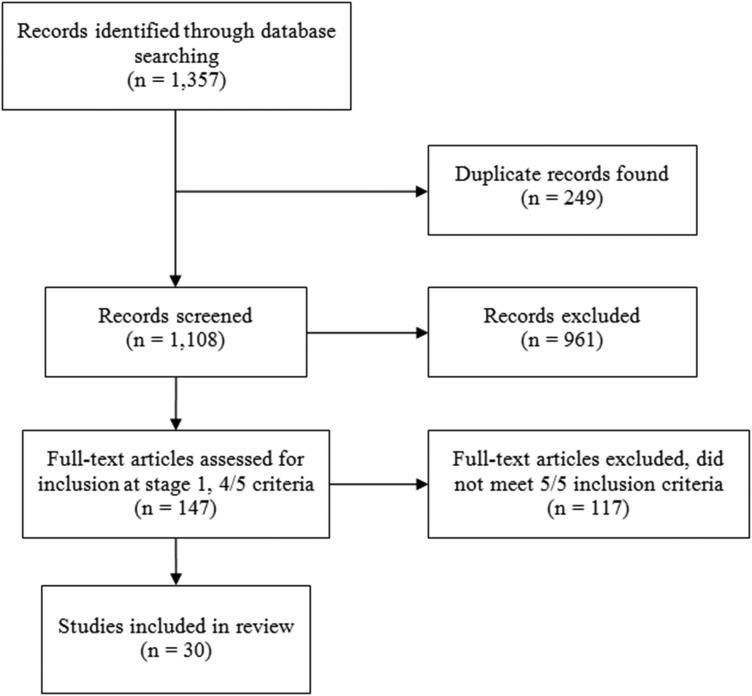
Search results.

From the 1357 papers identified, 817 were found through PubMed, 271 from Cochrane Library, and 269 from CINAHL. Removing 249 duplicates, 1108 papers were screened based on titles and abstracts. Of these, 147 met the criteria for the first stage of inclusion (four out of five criteria for inclusion met). Upon reviewing full texts, a final set of 30 papers were included although two of these reported on the same randomized controlled trial on MANAS in India (Patel *et al.* 2010*b*, 2011), and three were based on different perspectives of the community mental health programme in Ghana (Agyapong *et al.* 2015*a*, *b*, 2016). No studies were excluded on the basis of quality. See Fig. 1 for search outcomes.

Thirteen papers were qualitative evaluations using surveys, interviews, focus groups, action research, implementation research, or case study methodology (Ali *et al.* 2010; Petersen *et al.* 2011; Thurman *et al.* 2014; Agyapong *et al.* 2015*a*, *b*, 2016; Larson-Stoa *et al.* 2015; Lorenzo *et al.* 2015; Magidson *et al.* 2015; Nimgaonkar & Menon, 2015; Abas *et al.* 2016; Wright & Chiwandira, 2016). Twelve papers were randomized controlled trials (Ali *et al.* 2003; Baker-Henningham *et al.* 2005; Rahman *et al.* 2008; Kumakech *et al.* 2009; Patel *et al.* 2010*b*, 2011; Tomlinson *et al.* 2011; Chatterjee *et al.* 2014; Pradeep *et al.* 2014; Rotheram-Borus *et al.* 2015). Three papers were pre/post- or prospective cohort studies (Adam *et al.* 2012; Whiteford *et al.* 2013; Yaya Bocoum *et al.* 2013; Hung *et al.* 2014; Padilla *et al.* 2015). Two papers included economic evaluation (Buttorff *et al.* 2012; Chatterjee *et al.* 2014). All studies showed that task shifting for mental health was feasible and acceptable in the given contexts; however, perceptions of quality of care provided by lay providers remain uncertain (Patel *et al.* 2011; Petersen *et al.* 2011; Rotheram-Borus *et al.* 2015; Agyapong *et al.* 2016). A meta-analysis of outcome measures was not done as the interventions were diverse, conducted at multiple scales, and included qualitative evaluations of stakeholder perceptions. See Table 5 for characteristics of included studies.

**Table 5.** Characteristics of included studies

Author	Type of evaluation	Setting	Type of lay provider	Type of service provided	Sample size	Findings
Abas *et al.* (2016)	Case study: acceptability and implementation	Zimbabwe	Lay health worker (LHW)	LHWs carry out structured psychosocial assessment and a screen test, following it up with advice, discharge, problem-solving therapy (PST), or referral. Where clients are suffering from socioeconomic problems, LHWs may refer to income-generating projects also taking place in collaboration with the Friendship project	Six staff interviews; six patient interviews; five focus groups with 8–12 per group	A collaborative care intervention, including screening, PST and referral for depression and other CMDs is positively received by patients (happier, valued, less stigmatized, less lonely), rewarding for female community LHWs to deliver, and can be sustained over time at low cost. Sharing similar socioeconomic backgrounds with their clients enabled LHWs in establishing more productive relationships with their clients and improved service delivery
Agyapong *et al.* (2015*b*) (I)	Perceptions survey tool and qualitative analysis	Ghana	Community mental health workers (CMHWs) (includes community psychiatric nurses, community mental health officers and clinical psychiatry officers)	Different cadres of CMHWs support mental health work and refer to psychiatrists where necessary. Community Mental Health Officers (CMHOs), the least specialized cadre, are meant to detect cases and not diagnose or treat; however, due to workforce shortage, they often do both	Eleven psychiatrists, 26 health policy directors, 164 CMHWs	CMHWs are not seen as undermining the role of psychiatrists and find it easy to refer major cases; however, due to the shortage of psychiatrists and the geographic barriers, referrals do not always take place, making it necessary to both better train CMHWs for role clarity, and to increase the numbers of psychiatrist available for supervision. Over the 7-year period studied, LHWs were making fewer referrals as they had gained more confidence in the scope of their practice. CMHWs believe that patients and other healthcare workers have concerns about the quality of care they provide
Agyapong *et al.* (2015*a*) (II)	Perceptions survey tool and qualitative analysis	Ghana	CMHWs include community psychiatric nurses, community mental health officers and clinical psychiatry officers	CMHWs address conditions such as schizophrenia, psychosis, epilepsy, dementia, and other common mental illness. In addition, they perform health education tasks; reproductive and child health services; link to psychiatric services and patient advocacy regarding social services	164 CMHWs	CMHWs work beyond the scope of their practice and training, they provide financial assistance to patients, and sometimes fill in at regional hospitals for general medical consultations. Less than a quarter of CMHWs work closely with a psychiatrist. CMHWs do not increase nor undermine the work of psychiatrists. Community Mental Health Officers (CMHOs), meant to detect cases, are often treating and prescribing medicines, which is beyond the scope of their practice and should be addressed by either enhancing the scope of their training or ensuring availability of other cadres of health workers to cover tasks not meant to be covered by non-specialized health workers. CMHWs are integrated in Ghana's health system; however collaboration with traditional or religious healers is minimal, even though these stakeholders are important community sources of care seeking
Agyapong *et al.* (2016)	Perceptions survey tool and qualitative analysis	Ghana	CMHWs include community psychiatric nurses, community mental health officers and clinical psychiatry officers	CMHO training programmes introduced in 2010 to address the gap in mental health services. CMHOs have shorter training than other CMHWs and are not meant to diagnose, treat or prescribe medicines, but do so regularly due to shortages	Eleven psychiatrists, 29 heath policy directors, 164 CMHWs	There is a gap in training and supervision and a disconnect between what psychiatrists and health policy directors perceive to be available to CMHWs and what is available to them in reality. Many CHMWs are working beyond the scope of their practice with inadequate training and supervision afforded to them. Further investment in supervision and training is necessary
Ali *et al.* (2003)	Randomized controlled trial	Pakistan	Women briefly trained from the same community	Supportive, problem-solving counselling was provided to women with depression in their homes for eight sessions	124 depressed women	Based on AKUADS (Aga Khan University Anxiety and Depression Scale) score, there was a net reduction in anxiety and depression of 21% in the intervention arm
Ali *et al.* (2010)	Quasi-experimental action research	Pakistan	Women community health workers (CHWs)	CHWs would visit the home of new mothers to offer basic cognitive behavioural therapy, and provide supportive and problem-solving counselling. CHWs later discussed these with a clinical psychologist on a weekly basis. Those with more serious cases were referred for treatment. Instruction on healthy child-rearing practices was also provided	102 postpartum women with depression	AKUADS (Aga Khan University Anxiety and Depression Scale) scores dropped more for counselled *v.* not counselled group; however, both groups experienced declines in depression possibly due to general support provided in parenting and child-rearing practices
Baker-Henningham *et al.* (2005)	Randomized controlled trial	Jamaica	Community health aides	Community health aides visited mothers’ homes weekly for a half-hour, demonstrating activities that engaged both parent and child and supporting parenting competence; in addition, they provided counselling and problem solving even though these were not explicitly included in the intervention	139 mothers with undernourished children	Significant decline in depressive symptoms was reported in mothers receiving home visit with those receiving 40–50 visits benefitting the most (compared with fewer visits)
Bolton *et al.* (2014)	Randomized controlled trial	Thailand	Lay counsellors	Lay counsellors provided a Common Elements Treatment Approach (CETA) to Burmese survivors of imprisonment, torture and related trauma. Transdiagnostic interventions capitalize on commonalities across evidence-based treatments instead of having one particular focus, making them more response to cross cutting needs using decision rules and guidelines, with flexibility for contextual differences	247 participants (intervention *n* = 182; wait list controlled *n* = 165)	CETA participants experienced improvements in all outcomes, including depression, post-traumatic stress, functional impairment, anxiety, and aggression
Buttorff *et al.* (2012)	Economic evaluation	India	Lay health worker	Collaborative stepped care for CMDs using: (1) lay health workers in primary care settings trained to provide psychosocial services, (2) physicians already in the clinic, and (3) mental health specialists making monthly visits. Intensity of care provided was matched with severity of disorder to optimize human resource allocation. Subjects were taught stress reducing strategies and provided with tailored information and access to relevant networks and support organizations. Case management and proactive monitoring formed the basis of the intervention	1648 people with anxiety/depression	In public facilities, patient in the intervention arm showed improved health outcomes and lower time costs; health system costs were similar across intervention and control groups.
Chatterjee *et al.* (2014)	Randomized controlled trial & economic evaluation	India	CHWs	Collaborative community-based care: treatment plans, psychoeducational material to patients, adherence management, peer support, rehabilitation, health promotion for physical ailments, and network links to community agencies to address social, legal, and economic challenges. This package of services was delivered by CHWs in three phases: (1) the intensive engagement phase (0–3 months), including six to eight home visits made by CHWs; (2) the stabilization phase (4–7 months), with sessions delivered once every 15 days; and (3) the maintenance phase (8–12 months), with sessions delivered once a month	282 schizophrenic patients	Collaborative community-based care including supervised CHWs was more effective than facility based services for people with moderate to severe schizophrenia, especially for overall disability. No effect was observed for stigma. Costs were greater in intervention with a third attributed to supervision costs
Hung *et al.* (2014)	Prospective cohort study	South Africa	CHWs	Task shifting for screening of depression among pregnant women	361 postpartum women	The study demonstrated the feasibility of incorporating depression screening into CHWs’ routine workflow
Kumakech *et al.* (2009)	Cluster randomized controlled trial	Uganda	Peer group support with teachers as facilitators	AIDS counselling with two peer group sessions per week: share fears, worries and concerns, problem identification and problem solving	326 children aged 10–15 years (intervention group *n* = 159 orphans, control *n* = 167 orphans)	After adjusting for baseline scores, follow-up scores for the intervention group in comparison with controls showed significant improvement in depression, anger, and anxiety, but not for self-concept. This study demonstrated that peer-group support intervention decreased psychological distress, particularly symptoms of depression, anxiety and anger
Larson-Stoa *et al.* (2015)	Programme evaluation study through routine data collection	Indonesia	Paraprofessionals	Psychosocial group and individual counselling programme lasting 3 months with follow-up for victims of torture	178 participants	The results indicated the participants’ anxiety symptoms, depressive symptoms, somatic symptoms, and functioning improved from the intake to the follow-up. The programme appeared to have been effective in reducing participants’ symptoms and impairment in functioning
Lorenzo *et al.* (2015)	Qualitative (In-depth interviews, with an inductive and interpretative phenomenological approach used to analyse data)	South Africa, Botswana, and Malawi	Community disability worker (CDW)	Community-based rehabilitation involving: (1) integrated management of health conditions and impairments with a strong family focus, (2) negotiating disability-inclusive community development, and (3) coordinated and efficient intersectoral management systems for disability inclusion	Sixteen CDWs who had at least 5 years’ experience of disability-related work in a rural area	Three main themes with sub-categories emerged demonstrating the competencies of CDWs. First, integrated management of health conditions and impairments within a family focus comprised ‘focus on the functional abilities’ and ‘communication, information gathering and sharing’. Second, negotiating for disability-inclusive community development included four sub-categories, namely ‘mobilizing families and community leaders’, ‘finding local solutions with local resources’, ‘negotiating retention and transitions through the education system’ and ‘promoting participation in economic activities’. Third, coordinated and efficient intersectoral management systems involved ‘gaining community and professional recognition’ and the ability to coordinate efforts (‘it's not a one-man show’). The CDWs spoke of their commitment to fighting the inequities and social injustices that persons with disabilities experienced. They facilitate change and manage the multiple transitions experienced by the families at different stages of the disabled person's development
Magidson *et al.* (2015)	Implementation research / case study	Iraq	CHWs	Brief behavioural activation treatment for depression (BATD) was adapted with cultural modifications for low-literacy patient population and tailored training for non-specialist CHWs with little to no experience in behavioural therapies	Thirteen (11 CHWs, one study psychiatrist and one clinical supervisor). 107 patients received the intervention	Of the 107 patients that received the intervention, there was 72% retention, and they completed all of the nine sessions. Case 1: despite challenges the client responded well to BATD. Client noted positive changes in her personal life. Case 2: client noted positive changes in her personal life and this was noted by her family members. Intervention was found to be acceptable and effective at reducing depressive symptoms and improving functioning
Mendenhall *et al.* (2014)	Implementation research (focus group discussions, in-depth interviews)	Ethiopia, India, Nepal, South Africa, and Uganda	CHWs	Packages of care at the community level focused on early identification, awareness raising, stigma reduction, increasing demand for appropriate mental health care, and addressing continuing care and social and economic needs of people with priority mental disorders	Seventy-seven CHWs, 110 community members, 80 service users and caregivers, 113 primary health care workers, 39 specialists and policy makers (36 focus groups, 164 in-depth interviews)	Task sharing mental health services is perceived to be acceptable and feasible in these LMICs as long as key conditions are met: (1) increased numbers of human resources and better access to medications, (2) ongoing structured supportive supervision at the community and primary care levels, and (3) adequate training and compensation for health workers involved in task sharing
Murray *et al.* (2015*b*)	Randomized controlled trial	Zambia	Lay counsellors	Lay counsellor-provided trauma-focused cognitive behavioural therapy (TF-CBT) to address trauma and stress-related symptoms among orphans and vulnerable children	257 children (intervention group *n* = 131, control *n* = 126)	TF-CBT provided by lay counsellors decreased trauma and stress-related symptoms as measured by the UCLA Posttraumatic Stress Disorder Reaction Index and improved functional impairment for high levels of trauma
Murray *et al.* (2014)	Implementation / operations research	Iraq & Thai/Burma Border	Lay counsellors	The study explored the implementation of a CETA, a transdiagnostic intervention for adults with mood or anxiety problems developed specifically for use with lay counsellors as opposed to single focus on evidence based treatments for one treatment category. CETA is a new approach to training of lay counsellors using decision rules based on evidence to guide selection and sequencing of treatment elements, allowing for flexibility in individual symptom presentation	Thirty-four counsellors; five supervisors	Lay counsellors were able to adhere to fidelity of the intervention while also using qualitative research findings and feedback into implementation design to account for cultural and contextual differences. The CETA approach allows counsellors to treat and manage clients’ symptoms while handling comorbidities and providing decision tools to help determine selection, sequencing and dosing in culturally-sensitive ways. Support through an apprenticeship model (supervision) ensured fidelity
Neuner *et al.* (2008)	Randomized controlled trial	Uganda	Lay counsellors	Lay counsellors (trained refugees) carried out manualized narrative exposure therapy and flexible trauma counselling (two treatment arms compared with a no treatment group) in a refugee settlement in Uganda, trained in a 6-week course	277 Rwandan and Somali refugees	Over 6–9 months, refugees in the treatment arms had improved clinical and statistical scores on the post-traumatic stress diagnostic scale, also demonstrating improvements in physical health
Nimgaonkar & Menon (2015)	Implementation research and impact evaluation through survey, focus group, and routine data collection	India	Village health workers and health animators	Programme integrated into pre-existing comprehensive medical programme to identify and manage psychiatric disorders rapidly, comprehensively and sustainably. Village health workers and health animators followed up on activities cataloguing patients’ compliance, functionality and treatment regimen	The eligible Adivasi population was 13 345 at the beginning and 14 816 at the end of the programme	It was possible to train staff at all levels as the first step of an effort to integrate mental health into a comprehensive medical care programme that had previously focused solely on treatable acute and chronic medical disorders. The success of the programme is partly attributable to the pre-existing network of medical healthcare workers who were attuned to local cultural beliefs, the decentralization of healthcare and the mental health educational programmes. Surveys conducted before and after programme initiation also suggested improved knowledge, attitudes and acceptance of mental illness by the community. The annual per capita cost of the programme was 122.53 Indian Rupees per person per annum (USD 1.61)
Padilla *et al.* (2015)	Pre-/post-assessment	Argentina	Health agents	Annual training of health agents was instituted to better detect signs of mental illness and offer earlier treatment to reduce duration of untreated psychosis (DUP)	672 260 population of province studied over 7 years for DUP	Consecutive years of training of health agents to improve screening and detection of mental illness, when coupled with an effective system to refer cases to specialty care, correlates with reductions in DUP in new cases detected in a rural environment
Patel *et al.* (2010*b*)	Randomized controlled trial	India	Lay health counsellor	Collaborative stepped care intervention with lay health counsellor	2796 participants	Patients with ICD-10 CMDs were more likely to have recovered at 6 months of collaborative stepped care than the control. There was strong evidence of effect in public facility attenders and no evidence of effect in private facility attenders
Patel *et al.* (2011)	Randomized controlled trial	India	Lay health counsellor	Collaborative stepped care intervention with lay health counsellor	2796 participants	Prevalence of ICD-10 CMDs and the severity of symptoms of depression and anxiety in individuals attending public primary healthcare facilities with a CMD and in the subgroup of individuals with depression, over a 12-month period, was reduced using the MANAS collaborative stepped-care intervention led by lay health counsellors. Reduction in the risk of suicidal behaviours (plans or attempts) and disability days (days of no work or reduced work) and weaker effects on overall disability scores were also seen
Petersen *et al.* (2011)	Post-intervention process evaluation	South Africa, Uganda	CHWs	A common implementation framework using a multi-sectoral community collaborative, task-shifting and self-help approach was used across both countries as part of the Mental Health and Poverty Project (MHaPP): (i) reorientation of district management towards integrated primary mental healthcare; (ii) establishment of community collaborative multi-sectoral forums; (iii) task shifting, which entailed establishing an expert consultancy liaison mental health team and training of general PHC staff and CHWs or equivalents in identification, management and referral of mental disorders; and (iv) promotion of self-help groups at the community level	Qualitative process interviews with unspecified range of key stakeholders across both countries, focus group discussions, and use of meeting notes and observational data	Sensitization efforts were successful in allocating more resources to community mental health integration into primary care. Collaborative multi-sectoral forum was successful in mobilizing some extra resources to support mental health. Mental health training provided to CHWs strengthened their capacity to respond to psychosocial problems and related CMDs they encountered in their regular home visits. Further, referral pathways were strengthened in this programme. The common implementation framework supported both countries in successfully integrating mental health services into primary care even with different foci and resource availability across countries. However, task shifting was more successful in South Africa than in Uganda where resource limitations and inadequate mental health specialization from CHWs created bottle necks in service delivery and demoralized CHWs. It is therefore important to ensure that the system has safeguards in place to support task shifting
Pradeep *et al.* (2014)	Randomized controlled trial	India	CHWs	Enhanced care by CHWs was provided to patients. CHWs visited patients immediately following the first medical consultation, educated the patient and her family members about depression and its treatment. This was followed by emphasis on adhering to treatment and medication regimen and at least four CHW visits as well as monthly physician consultation	260 adults with depression	Seeking and adhering to treatment was higher in the intervention group; however, there was no significant difference in severity of depression or quality of life between groups or between completers and dropouts at six months.
Rahman *et al.* (2008)	Cluster randomized controlled trial	Pakistan	Lady Health Workers	Trained lady health workers held a weekly session that included cognitive behavioural therapy for 4 weeks in the last month of pregnancy, three sessions in the first postnatal month, and nine 1-monthly sessions thereafter	1054 pregnant women	Integration of a cognitive behaviour therapy-based intervention into the routine work of CHWs more than halved the rate of depression in prenatally depressed women compared with those receiving enhanced routine care. In addition to symptomatic relief, the women receiving the intervention had less disability and better overall and social functioning, and these effects were sustained after 1 year
Rotheram-Borus *et al.* (2015)	Cluster randomized controlled trial	South Africa	Mentor Mothers (CHWs)	Building on its existing home-visiting programme: CHWs were trained for 1 month in cognitive behavioural change strategies and role-playing. They were trained to provide and apply health information about general maternal and child health, HIV/TB, alcohol use, and nutrition to low-income, urban women's lives	1238 mothers	Despite not originally targeting reductions in maternal depression or improved maternal emotional health, the home-visiting intervention with urban South African mothers was associated with improved maternal emotional health 36 months after their children were born. CHWs encouraged and trained mothers to care for their infants, regardless of stress. Relative to standard care, intervention mothers were significantly less likely to report depressive symptoms and more positive quality of life at 36 months. Alcohol use was significantly related to use over time, but was also related to depression and HIV status at each assessment and associated with partner violence at 36 months. A more intensive and group-focused intervention is needed to address alcohol use
Thurman *et al.* (2014)	Longitudinal quasi-experimental design: pre/post-assessment	South Africa	Lay volunteers and trained paraprofessionals	Two models were tested: (1) home-visiting programmes that use a trained and compensated paraprofessional workforce and (2) programmes that rely on volunteers, who most often receive limited training and nominal incentives for their efforts.	1487 children and 918 caregivers	No measurable reduction in psychological distress among children or caregivers served by paraprofessionals compared to volunteers was observed. Child behavioural problems, depression among boys, and family functioning were worse by follow-up, regardless of programme model
Tomlinson *et al.* (2011)	Cluster randomized controlled trial	South Africa	Local women trained as CHWs	Local women with good social skills (and mothers themselves) carried out the Philani intervention Programme, which consists of home visits with pregnant women and interventions to reduce alcohol misuse, increase adherence to perinatal HIV regimens, and boost child nutrition. CHWs were trained in: (1) cognitive-behavioural approaches to establishing healthy routines and to problem-solving around goal setting, choices, triggers, and shaping of desirable behaviours; (2) key information about general maternal and child health, techniques for framing each health issue that is a risk (nutrition, alcohol, and HIV), and strategies for applying the health information in families’ daily lives; and (3) coping with their own life challenges	1238 pregnant women	Training CHWs as generalists appears to benefit child growth by preparing them to address the highest priority health issues, to address general maternal and child health, and to practice effective caretaking and problem solving.
Wright & Chiwandira (2016)	Impact evaluation of scale-up and integration	Malawi	Health surveillance assistants (HSAs)	The intervention involved four elements: (1) reducing clients’ risk of harm to self or others, (2) providing client-focused psychoeducation, (3) providing clients with psychological and emotional support, and (4) promoting psychosocial support through families and the wider community	224 people in distress	HSAs’ approach to mental health care delivery was found to be both credible and practical in meeting the needs of the population. Sustained scale-up and integration of the delivery model was observed. Increased case detection was seen by HSAs. No changes were observed in visits to psychiatric hospitals

Studies were conducted in India, Ghana, Zimbabwe, Pakistan, Malawi, South Africa, Uganda, Indonesia, Iraq, Argentina, Botswana Jamaica, Ethiopia, Zambia, and Thailand, primarily at the district (includes village) level (see Table 5). Across these different contexts, community mental health programmes were variable in nature with some being more integrated into existing health systems (Patel *et al.* 2010*b*, 2011; Petersen *et al.* 2011; Mendenhall *et al.* 2014; Agyapong *et al.* 2015*a*, *b*; Nimgaonkar & Menon, 2015; Agyapong *et al.* 2016; Wright & Chiwandira, 2016). Others were more programme-specific in nature and targeted specific at-risk populations, such as mothers suffering from depression, people living with HIV/AIDS, orphans, refugees and torture survivors (Baker-Henningham *et al.* 2005; Neuner *et al.* 2008; Kumakech *et al.* 2009; Ali *et al.* 2010; Bolton *et al.* 2014; Murray *et al.* 2014; Larson-Stoa *et al.* 2015; Magidson *et al.* 2015; Murray *et al.* 2015*b*). Outcome measures used included mental health assessment tools, such as the 10-item Edinburgh Postnatal Depression Scale (EPDS-10), the Center for Epidemiological Studies Depression Scale (CES-D), Psychiatric Symptom Score, UCLA Post-Traumatic Stress Disorder Reaction Index, Aga Khan University Anxiety and Depression Scale (AKUADS), and the Positive and Negative Syndrome Scale (PANSS). Qualitative measures of impact included participatory action research, implementation research, case study, and other qualitative approaches with an aim to explore broader systems components.

### Intervention effects across the building blocks

Of the 30 studies, 25 (83%) included mention of the six WHO health system building blocks other than service delivery and health workforce (Table 6). All 30 studies included some aspect of the seventh additional building block (communities and people) through community engagement and/or efforts to understand community needs in order to best integrate lay providers.

**Table 6.** System building blocks mentioned in each study

Author	Building blocks	Barriers across building blocks	Facilitators across building blocks	Systems thinking characteristics	Intersectoral collaboration
Abas *et al.* (2016)	SD, HRH, IT, FS, C	Financial incentives for lay providers; payment mechanisms for patients. Data management tools need improvement	Links to income-generation projects for patients		
Agyapong *et al.* (2015*b*) (I)	SD, HRH, IT, C	Poor documentation			
Agyapong *et al.* (2015*a*) (II)	SD, HRH, IT, MD, FS, LG, C	Lack of training in psychotropic medicine & inappropriate prescribing practice; demand-side financing	Involvement of key policy stakeholders increased understanding of ground level realities; support from mental health professionals; collaboration with traditional healers	Identification of stakeholder perspectives	
Agyapong *et al.* (2016)	SD, HRH, IT, MD, FS, LG, C	Perceptions of quality; inappropriate prescribing practice; lack of financing to facilitate access by patients; disconnect with policy makers	Involvement of policy stakeholders; Mobile technology for supervision	Identification of stakeholder perspectives	
Ali *et al.* (2010)	SD, HRH, MD, C		Acceptability enhanced due to resistance of women to use of pharmacotherapy		
Bolton *et al.* (2014)	SD, HRH, HIS, C		Step sheets used to ensure fidelity and follow-up	To better serve the psychosocial needs of the population, ‘the apprenticeship model included feedback loops encouraging local counsellors and supervisors to modify delivery of components to increase the fit with the culture and local setting, based on their ongoing experiences’	The trial is a collaboration across NGOs: Burma Border Projects (an international NGO), and three local service organizations – Assistance Association for Political Prisoners–Burma (AAPP), Mae Tao Clinic (MTC), and Social Action for Women (SAW), funded by US Agency for International Development Victims of Torture Fund
Buttorff *et al.* (2012)	SD, HRH, FS, C	Determining cost to households of mental illness is difficult due to the variable ways households cope with illness	Scale-up found to be cost effective based on model proposed		
Chatterjee *et al.* (2014)	SD, HRH, MD, FS, C		Caregivers enhanced adherence to medicines; social and economic recovery were identified as important contributors to mental health interventions; support provided for access to employment opportunities		Established ‘networks with community agencies to address social issues, to help with social inclusion, access to legal benefits, and employment opportunities’.
Hung *et al.* (2014)	SD, HRH, IT, C	Heavy workloads	Technology for screening		
Larson-Stoa *et al.* (2015)	SD, HRH, FS, C	Gender differences in treatment response; unable to provide care to all (psychosis patients) due to financial limitations			
Lorenzo *et al.* (2015)	SD, HRH, IT, MD, FS, LG, C	Lack of horizontal coordination across different sectors involved in disability management	Referral management systems; financial advice to patients	Identification of lack of coordination across sectors working on disability and associated feedback mechanism	Education, Social Development, Transport sectors involved; lack of coordination was a challenge
Magidson *et al.* (2015)	SD, HRH, IT, C		Telemedicine for supervision		
Mendenhall *et al.* (2014)	SD, HRH, IT, MD, FS, LG, C	Lack of infrastructure, overburdening workload, community preferences around who should work as lay providers, lack of recognition for taking on new roles, unclearly defined roles, lack of private spaces for mental health consultation, and confidentiality; social and educational factors posed challenges to acceptability (i.e. perceived inability to provide sufficient care); lack of transport to a health facility, inadequate compensation, and limited availability of specialists for training and supervision of lay providers; failure to prioritize psychotherapy and behavioural interventions alongside a bias toward medication		Identification of stakeholder perspectives, systemic challenges, and sociocultural nuances	
Murray *et al.* (2015*b*)	SD, HRH, FS, LG, C		Workload and retention; lack of funding (minimal sessions and only one post assessment follow-up		Where high-risk cases were identified, the Child Protection Unit was informed, initiating an investigation for child abuse and neglect
Murray *et al.* (2014)	SD, HRH, IT, C	Transportation, personnel problems, culture and climate, and buy-in	Apprenticeship model using step sheets and detailed information allowed the project to work	Barriers and facilitators identified during the implementation of the project were fed back into implementation design, adjusting for cultural and contextual needs (e.g., addition of alcohol use)	
Neuner *et al.* (2008)	SD, HRH, FS, LG, C	Forced repatriation in settlement camps forced refugees into hiding and a resettlement programme caused loss to follow-up; basic package of health services did not include mental health			Access to food, economic situation and educational background were captured to provide sociodemographic background. The Ugandan government, the red cross, and the United Nations High Commissioner for refugees provided basic package of health services, and food packages, respectively
Nimgaonkar & Menon (2015)	SD, HRH, IT, MD, FS, LG, C	Medicines shortages; demand-side financial barriers	Decentralization of mental health services		Education sector involved
Padilla *et al.* (2015)	SD, HRH, IT, FS, C		Technology for screening; provincial system's universal coverage mechanism		
Patel *et al.* (2010*b*)	SD, HRH, IT, MD, FS, LG, C	Perceptions of quality of care; prescribing practice and access to medicines	Telemedicine for supervision		
Patel *et al.* (2011)	SD, HRH, MD, LG, C	Prescribing practice and access to medicines	Person-centred approach in private facilities showed similar effects to the collaborative care approach		
Petersen *et al.* (2011)	SD, HRH, IT, MD, FS, LG, C	Shortage of medicines	Supporting socioeconomic wellbeing in patients (improve financial access); decentralization of mental health services	Links made across sectors and description of these interactions	Multi-sectoral forum; Agriculture sector
Rotheram-Borus *et al.* (2015)	SD, HRH, IT, C		Training in documentation on mobile phones		
Thurman *et al.* (2014)	SD, HRH, IT, FS, C	Effective resources allocation across community resources	Embedding monitoring and evaluation in programme design		Multiple sectors necessary to achieve treatment effect not seen in intervention
Tomlinson *et al.* (2011)	SD, HRH, IT, C		Mobile supervision technology.		
Wright & Chiwandira (2016)	SD, HRH, MD, C		Training in supporting management of medication		Health promotion activities took place all over the community including in schools and churches

SD, Service delivery; HRH, Health workforce; IT, Information and Technology; MD, Medicines & Medical Devices; FS, Financing Systems; LG, Leadership & Governance; C, Community.

Sixteen studies of the 25 (80%), considered the role of information and technology. This building block was often mentioned in terms of use of technology for screening of mental illness (Hung *et al.* 2014; Padilla *et al.* 2015), use of mobile technology for supervision of lay providers (Tomlinson *et al.* 2011; Magidson *et al.* 2015; Agyapong *et al.* 2016), and need for improved data management tools to ensure adequate follow-up patients at-risk of poor mental health (Agyapong *et al.* 2015*b*; Abas *et al.* 2016). Facilitators identified to support this need were the use of step sheets for enhanced fidelity to interventions and training on documentation of patient visits on mobile phones (Bolton *et al.* 2014; Rotheram-Borus *et al.* 2015; Murray *et al.* 2015*b*).

Eleven studies (55%) considered the implications of the medicines and medical devices. The discrepancies between training and service delivery in prescribing practices were a challenge in task shifting for mental health (Agyapong *et al.* 2016). That is, lay providers, not trained in prescription of psychotropic medicines, found themselves prescribing them due to community needs (Agyapong *et al.* 2015*a*). Shortage of medicines and the resulting limitations placed on lay providers were impediments in achieving improved health outcomes and demoralized providers who were unable to provide adequate care (Petersen *et al.* 2011; Nimgaonkar & Menon, 2015). The bias towards medication as treatment also created challenges for prioritization of psychotherapy and behavioural interventions, affecting demand-side acceptability (Mendenhall *et al.* 2014).

Fifteen studies (70%) raised financing issues in task shifting for mental health with most referring to lack of funds as a limitation to scale-up, pointing to the need to prove cost-effectiveness as a means of ensuring investment by policy and decision makers (Agyapong *et al.* 2015*a*, 2016; Murray *et al.* 2015*b*). Lack of financial incentives for lay providers and their supervisors was another challenge raised (Mendenhall *et al.* 2014; Abas *et al.* 2016). Some studies mentioned demand-side financing as a barrier to improved mental health delivery, citing the ability to pay for basic mental health services from the patient's perspective (Neuner *et al.* 2008; Nimgaonkar & Menon, 2015; Abas *et al.* 2016). Ensuring that referrals were made to services covered by social protection mechanisms, was raised as an important element of providing sustainable and effective mental health service delivery by lay providers (Lorenzo *et al.* 2015; Padilla *et al.* 2015). Supporting patients through advice for socioeconomic well-being and links to income-generating projects was a means through which lay providers tried to address demand-side financial barriers (Baker-Henningham *et al.* 2005; Petersen *et al.* 2011; Lorenzo *et al.* 2015).

Finally, 10 studies (50%) mentioned leadership and governance issues with reference to task shifting for mental health. Programme-level supervision of lay providers, which was raised as a challenge across most of the studies included, was not captured as an overarching leadership and governance issue in this review as it is not sufficiently addressing system-level leadership and governance (Schneider & Lehmann, 2016). Perception surveys in Ghana directly involved policy directors, which provided an improved understanding of the gap between perceptions of lay provider programmes by policy directors and realities in the field (Agyapong *et al.* 2015*a*, *b*, 2016). Other studies referenced the need for policy support to integrate mental health services by lay providers into existing practice, citing governance structures as facilitators in scale-up and integration of mental health services through decentralization of these services (Petersen *et al.* 2011; Nimgaonkar & Menon, 2015). Leadership and governance structures were also barriers to integration. In larger, multi-country studies, lack of clarity in lay provider roles and confidentiality issues undermined integration of programmes from a supply-side perspective (Mendenhall *et al.* 2014). Community-level acceptability of programmes and perceptions on who can be a lay provider were cited as demand-side challenges that need mitigation through improved transparency, accountability, and leadership that listens to the needs of the population, such as the need for transportation (Mendenhall *et al.* 2014). One study highlighted the siloed effect of multiple vertical programmes addressing disability across different sectors with no oversight or horizontal coordination (Lorenzo *et al.* 2015). In programmes targeted at vulnerable populations, such as refugees and orphans, continuity was a challenge as these populations are mobile. Leadership and governance issues beyond the health sector played a heavy role in the ability of lay providers to provide necessary mental health services; therefore, collaboration with other officials was raised as being important to the intervention (Neuner *et al.* 2008; Murray *et al.* 2015*b*).

### The use of systems thinking tools in evaluation of interventions

System dynamics theory or modelling tools were not directly used in any of the included studies; however, six studies took a more comprehensive approach in capturing system implications of the intervention being studied. An important element of systems thinking is understanding roles, characteristics, and interactions of the players involved. The perceptions surveys conducted in Ghana, the phenomenological approach across South Africa, Botswana and Malawi, the multi-country stakeholder perspective mapping, and the cross-country comparison of South Africa and Uganda through interviews and focus groups captured such perspectives and allowed for improved understanding of gaps to ensure successful scale-up and integration into the health system (Petersen *et al.* 2011; Mendenhall *et al.* 2014; Agyapong *et al.* 2015*a*, 2016; Lorenzo *et al.* 2015). These studies demonstrated the range of actors necessary for successful integration and showed that actors may have different interpretations of challenges, and different strengths in mitigating these challenges. Systems thinking also should allow for a non-linear process of change, whereby study findings are fed back into the design of the intervention; implementation research methods facilitate this, making adjustments for context and cultural needs (Mendenhall *et al.* 2014; Murray *et al.* 2014; Nimgaonkar & Menon, 2015).

In community mental health, robust referral pathways are an important piece of integration and working across stakeholders is necessary to ensure appropriate follow-up and service delivery for patients, not just within the health system, but also across other social sectors (Petersen *et al.* 2011; Lorenzo *et al.* 2015). Intersectoral components of included studies were captured in this review where available. Intersectoral collaboration here is based on the WHO concept of intersectoral action for health, defined as ‘a recognised relationship between part or parts of the health sector with parts of another sector which has been formed to take action on an issue to achieve health outcomes (or intermediate health outcomes) in a way that is more effective, efficient or sustainable than could be achieved by the health sector acting alone’ (WHO, 1997). Eight studies touched on efforts made beyond the health sector. These interventions focused on the education sector, where peer group support for AIDS counselling (Kumakech *et al.* 2009) or support for disability management (Lorenzo *et al.* 2015) would take place; across non-governmental organizations for vulnerable populations (Bolton *et al.* 2014); and with the criminal and social services sectors (Murray *et al.* 2015*b*). In addressing disability challenges, social development and transport sectors were involved to make the lived environment more supportive of those living with both physical and mental disabilities (Lorenzo *et al.* 2015). Collaboration with the judicial system was also important in cases where abuse and neglect were part of the diagnosis (Murray *et al.* 2015*b*).

Several studies raised social determinants of mental health, such as socioeconomic status, employment, lack of education, and violence as risk factors that needed to be addressed in order to enhance the positive effect of task shifting for mental health (Petersen *et al.* 2011; Mendenhall *et al.* 2014; Thurman *et al.* 2014; Lorenzo *et al.* 2015; Nimgaonkar & Menon, 2015; Wright & Chiwandira, 2016). One study highlighted health promotion activities through working with community resources, such as schools and churches, as an enabling factor (Wright & Chiwandira, 2016). Another mentioned the lack of such collaboration with other sectors as a barrier in seeing improved treatment outcomes (Thurman *et al.* 2014). Four studies had formal arrangements for embedding intersectoral practice in the task shifting (Petersen *et al.* 2011; Lorenzo *et al.* 2015). The intersectoral fora created to support these programmes strengthened their ability to integrate into existing systems and provided a wider range of community referral pathways for lay providers to use in linking their patients to the resources necessary for thriving, thereby indirectly enhancing mental health (Petersen *et al.* 2011; Chatterjee *et al.* 2014; Lorenzo *et al.* 2015). One such example is the referral of patients to income-generating programmes within the agricultural sector (Petersen *et al.* 2011).

## Discussion

Despite the global call to action to improve scale-up and integration of lay provider programmes, the evidence base around implementation, scale-up, and integration of task-shifting strategies for mental health remains limited in both quantity and breadth (GHWA, 2013; Weinmann & Koesters, 2016). Moving from fragmentation to integration requires a move beyond issues specific to lay provider programmes, such as remuneration, training, and supervision (Schneider & Lehmann, 2016). It needs an understanding of large-scale public sector involvement, interactions across key actors, mobilization of these actors, and monitoring and evaluation tools that capture the complex adaptive parts within the system as they shift and respond to scale-up toward a true community system (Hanlon *et al.* 2014; Schneider & Lehmann, 2016). A systems thinking approach can help capture these complexities and understand how to optimize community mental health systems (Peters, 2014).

This review demonstrates that there is space for more systematic approaches to studying health systems elements that affect and/or are impacted upon by task-shifting interventions for mental health. None of the included studies systematically studied system elements; however, many touched upon the WHO building blocks of the health system other than those directly related to task shifting (i.e. service delivery and health workforce). These studies included qualitative methods that allowed them to capture some of the interactions within the system and highlight barriers, facilitators and effects that fell outside the limited scope of the task-shifting intervention (Petersen *et al.* 2011; Lorenzo *et al.* 2015; Nimgaonkar & Menon, 2015; Padilla *et al.* 2015; Agyapong *et al.* 2016).

### Barriers and facilitators of scaling up mental health care by the building blocks

Barriers to scaling up mental health services identified across studies included: stigma around mental health in the community (Ali *et al.* 2010; Nimgaonkar & Menon, 2015; Padilla *et al.* 2015); poor documentation and loss of follow-up due to lack of robust data management and patient management tools (Agyapong *et al.* 2015*b*); lack of access to psychotropic medicines and/or lack of sufficient training for rational prescribing practice (Patel *et al.* 2011; Agyapong *et al.* 2015*a*; Nimgaonkar & Menon, 2015); geographic and financial demand-side barriers to access of mental health services (Baker-Henningham *et al.* 2005; Petersen *et al.* 2011; Mendenhall *et al.* 2014; Agyapong *et al.* 2015*a*); poor collaboration with spiritual and traditional healers (Agyapong *et al.* 2015*a*); disconnect between providers and decision makers (Agyapong *et al.* 2015*a*, 2016; Rotheram-Borus *et al.* 2015); existing heavy workload of lay providers (Petersen *et al.* 2011; Hung *et al.* 2014); gender differences in responding to treatment (Larson-Stoa *et al.* 2015); and lack of access to community resources to support social determinants of mental health (Tomlinson *et al.* 2011; Thurman *et al.* 2014; Rotheram-Borus *et al.* 2015).

Facilitators to scaling up mental health services identified across studies included: suitability of lay providers due to their ability to relate to the community and their patients (Baker-Henningham *et al.* 2005; Padilla *et al.* 2015; Abas *et al.* 2016); support from specialized mental health professionals (Agyapong *et al*. 2015*a*, *b*); use of technology and telemedicine to support supervisory practice (Patel *et al.* 2011; Tomlinson *et al.* 2011; Magidson *et al.* 2015; Agyapong *et al.* 2016); integrated interventions that include life skill building for sustainable livelihood practice, social interaction, and self-care (Petersen *et al.* 2011; Chatterjee *et al.* 2014); and integration into existing networks with robust service delivery models that support lay providers (Petersen *et al.* 2011; Nimgaonkar & Menon, 2015).

While information and technology tools appear to be facilitators for optimizing service delivery by lay providers, care must be taken in the selection of technology solutions. It is critical to understand how providers use technology as a part of the system. Some tools require the interpretation and training of health professionals to be appropriately and efficiently used, suggesting that not all technologies are readily transferable across health workforce cadres (Jotheeswaran *et al.* 2015; Robbins *et al.* 2015). Inefficiencies in the system can also be found in poor access to medicines (WHO, 2009). Financial and procurement barriers impede access to essential psychotropic medicines, impeding the delivery of appropriate mental health care to those who require pharmacotherapy (Agyapong *et al.* 2015*a*; Nimgaonkar & Menon, 2015). Scaling up mental health treatment by lay providers without addressing access to medicines in parallel will prove unsuccessful by undermining the quality and impact of additional service provision (WHO, 2009).

The barriers and facilitators outlined here showcase the complexity involved in task shifting for mental health and the need for a broader systems approach to mitigating barriers and leveraging facilitators. By being community-based and having a deep understanding of community needs and assets, lay providers have an enhanced ability to identify social determinants of mental health within a given context (Richters *et al.* 2013; Padilla *et al.* 2015). This rich knowledge, combined with appropriate training, puts them in the optimal position to refer patients to relevant social services (Paudel *et al.* 2014). Mental health is often a comorbidity in chronic disease management; training programmes should also prepare lay providers with the knowledge and skills necessary to understand such linkages and to refer appropriately (Rotheram-Borus *et al.* 2015).

Establishing networks and intersectoral linkages is not easy. Despite policy support, implementation and scale-up of integrated approaches to strengthening community mental health remains a challenge (Hanlon *et al.* 2014). Even where formal mechanisms are in place for intersectoral collaboration (i.e. where formal engagement of health, education and development sectors are embedded in programme design), participation was erratic and uncertain without senior officials present (Petersen *et al.* 2011). Existing models and formal arrangements of intersectoral collaboration require additional incentives and governmental support. In this way, partnerships move beyond platitudes and truly work as collaborative fora that support lay providers in assessing patient needs and selecting appropriate referral pathways.

### Implications for future research

Mental health is rarely an isolated problem. It sometimes stems from physical, environmental, or sociocultural challenges and creates positive feedback loops that become difficult to break (Tomlinson *et al.* 2011; Thurman *et al.* 2014). Taking a systems thinking approach to unpacking task-shifting interventions for mental health will unveil extant opportunities and threats in the current system. A system-level understanding of interventions will allow for improved integration and effective engagement of important actors overlooked in the traditional model of implementation and evaluation. Such actors include caregivers, non-governmental entities that support social determinants of mental health, employers, spiritual leaders, and other social sectors (e.g., education, agriculture, transport, social development, etc.) (Schneider & Lehmann, 2016). Representing the system overall and opportunities for improvement in the implementation and evaluation of such programmes can advocate for further investment in community mental health systems strengthening.

With appropriate evidence describing the roles and contributions of diverse sectors to mental health outcomes, there is potential to facilitate strategic intersectoral investment for optimal health impact as well as cost-effectiveness. Stigma, for instance, is an often-cited barrier in mental health seeking behaviours and even in provision of mental health care (van Ginneken *et al.* 2013; Nimgaonkar & Menon, 2015; Iheanacho *et al.* 2016; Weinmann & Koesters, 2016). A study of church-based lay providers showed that higher education was correlated with improved bio-psychosocial perspectives on mental health and fewer displays of stigma-based behaviour (Iheanacho *et al.* 2016). Overcoming stigma is therefore not necessarily limited to the role of the health sector; the education sector can play an important part in addressing stigma by talking about mental health and raising awareness.

Due to the nature of the search strategy, this review highlighted interventions that were conducted primarily in the health sector. Few included studies employed task-shifting strategies across other sectors to enhance mental health promotion. More examples of such collaboration exist, especially in education and social services. Therefore, it would be worth conducting a more targeted review of the evidence on interventions happening in other sectors that have impacts on mental health. Skill-mixing interventions also warrant more emphasis as they highlight the need for a range of skills beyond the health sector to address mental health challenges. Comparing the effectiveness of mental health-related interventions housed in the health sector *v.* those in others would be valuable in identifying cost-effective opportunities for intersectoral collaboration and cohesive strategies for mental health.

Limitations of this review may include the wide variety of mental health interventions, populations studied, and outcome measures included, making it potentially difficult or inappropriate to apply this review's broader conclusions to unique mental health conditions. While the majority of studies did not explicitly use a systems thinking approach, some studies indicated implicit consideration of systems thinking characteristics. It is possible that studies neglecting to mention system-wide effects in final manuscripts did in fact acknowledge these effects in the design and implementation of interventions to some degree, but this data was subsequently not available for this review.

## Conclusions

Task shifting for mental health has been demonstrated as an acceptable and effective approach to addressing the mental health gap in LMICs. This review shows the complexity of task-shifting interventions by exploring interactions of intervention elements and actors across the six WHO building blocks. There is a lack of systematic approaches to exploring this complexity in the evaluation of task-shifting interventions. Systems thinking tools should support evidence-informed decision making for a more complete understanding of community-based systems strengthening interventions for mental health.

## Annexure 1. Search Strategy

### CINAHL-Ebsco

#### Concept 1. Mental Health

(MH “Mental Health Personnel”) OR (MH “Mental Health”) OR (MH “Mental Health Services”) OR (MH “Community Mental Health Services+”) OR (MH “Community Mental Health Nursing”) OR (MH “Mental Health Organizations”) OR (MH “Developmental Disabilities”) OR (MH “Intellectual Disability”) OR (MH “Health Services for Persons with Disabilities”) OR (MH “Mental Disorders”) OR (MH “Support, Psychosocial”) OR (MH “Depression”) OR (MH “Bipolar Disorder”) OR (MH “Dementia”) OR (MH “Schizophrenia”) OR MH “substance use disorders” OR TI “mental health” or TI “mental healthcare” or TI “mental illness” or TI “mental disorder” or TI “mental disorders” or TI “disabled” or TI “disability” or TI “disabilities” or TI “neurologic disorder” or TI “depression” or TI “depressive” or TI “depressed” or TI “PTSD” or TI “psychosis” or TI “psychoses” or TI “psychotic” or TI “Schizophrenia” or TI “bipolar” or TI “epilepsy” or TI “seizures” or TI “Developmental Disabilities” or TI “Learning Disorders” or TI “autism” or TI “autistic” or TI “dementia” or TI “substance abuse” or TI “overuse” or TI “substance dependence” or TI “drug dependency” or TI “harmful use” or TI “hazardous use” or TI “suicide” or TI “self-harm” or TI “mental retardation” or TI “neurotic” or TI “Alcoholism” or TI “alcoholic” or TI “psychotropic” or TI “anxiolytics” or TI “depressant” or TI “epileptic” or TI “mood stabilizers” or TI “psychosocial support” or TI “psychology” or TI “psychological” or TI “psychotherapy” or TI “rehabilitation” or TI “stigma” or TI “support group” or TI “cognitive therapy” OR TI “reality therapy” OR TI “behavior therapy” or TI “behaviour therapy” or TI “self-help group” OR AB “mental health” or AB “mental healthcare” or AB “mental illness” or AB “mental disorder” or AB “mental disorders” or AB “disabled” or AB “disability” or AB “disabilities” or AB “neurologic disorder” or AB “depression” or AB “depressive” or AB “depressed” or AB “PTSD” or AB “psychosis” or AB “psychoses” or AB “psychotic” or AB “Schizophrenia” or AB “bipolar” or AB “epilepsy” or AB “seizures” or AB “Developmental Disabilities” or AB “Learning Disorders” or AB “autism” or AB “autistic” or AB “dementia” or AB “substance abuse” or AB “overuse” or AB “substance dependence” or AB “drug dependency” or AB “harmful use” or AB “hazardous use” or AB “suicide” or AB “self-harm” or AB “mental retardation” or AB “neurotic” or AB “Alcoholism” or AB “alcoholic” or AB “psychotropic” or AB “anxiolytics” or AB “depressant” or AB “epileptic” or AB “mood stabilizers” or AB “psychosocial support” or AB “psychology” or AB “psychological” or AB “psychotherapy” or AB “rehabilitation” or AB “stigma” or AB “support group” or AB “cognitive therapy” OR AB “reality therapy” OR AB “behavior therapy” or AB “behaviour therapy” or AB “self-help group”

#### Concept 2. Community health workers

(MH “Community Health Workers”) OR (MH “Rural Health Personnel”) OR MH “Allied Health Personnel” (MH “Community Health Services”) OR TI “health extension worker” or TI “health extension workers” or TI “community health worker” or TI “community health workers” or TI “community health aide” or TI “home health aide” or TI “community health representative” or TI “community health representatives” or TI “community networks” or TI “peer group” or TI “lay volunteer” or TI “lay worker” or TI “lay health worker” or TI “lay health workers” or TI “lay health advisor” or TI “lay health advisors” or TI “barefoot doctor” or TI “barefoot doctors” or TI “peer to peer” or TI “community based practitioner” or TI “community based practitioners” or TI “community-based practitioner” or TI “community-based practitioners” or TI “Accredited social health activist” or TI “Accredited social health activists” or TI “village health worker” or TI “village health workers” or TI “village health guide” or TI “village health guides” or TI “village health support guide” or TI “village health support guides” or TI “health auxiliary worker” or TI “health auxiliary workers” or TI “front-line health worker” or TI “front-line health workers” or TI “Shasthyo Sebikas” or TI “Community Outreach Worker” or TI “Community Outreach Workers” or TI “Peer Counsellor” or TI “Peer Counsellors” or TI “Peer Counselour” or TI “Peer Counselours” or TI Promotora or TI “peer educator” or TI “peer educators” OR TI “non-physician healthcare worker” OR TI “non-physician healthcare workers” or TI “task-shifting” or TI “task shifting” or TI “task-sharing” OR AB “health extension worker” or AB “health extension workers” or AB “community health worker” or AB “community health workers” or AB “community health aide” or AB “home health aide” or AB “community health representative” or AB “community health representatives” or AB “community networks” or AB “peer group” or AB “lay volunteer” or AB “lay worker” or AB “lay health worker” or AB “lay health workers” or AB “lay health advisor” or AB “lay health advisors” or AB “barefoot doctor” or AB “barefoot doctors” or AB “peer to peer” or AB “community based practitioner” or AB “community based practitioners” or AB “community-based practitioner” or AB “community-based practitioners” or AB “Accredited social health activist” or AB “Accredited social health activists” or AB “village health worker” or AB “village health workers” or AB “village health guide” or AB “village health guides” or AB “village health support guide” or AB “village health support guides” or AB “health auxiliary worker” or AB “health auxiliary workers” or AB “front-line health worker” or AB “front-line health workers” or AB “Shasthyo Sebikas” or AB “Community Outreach Worker” or AB “Community Outreach Workers” or AB “Peer Counsellor” or AB “Peer Counsellors” or AB “Peer Counselor” or AB “Peer Counselors” or AB “Peer Counselour” or AB “Peer Counselours” or AB Promotora or AB “peer educator” or AB “peer educators” OR AB “non-physician healthcare worker” OR AB “non-physician healthcare workers” or AB “task-shifting” or AB “task shifting” or AB “task-sharing”

#### Concept 3. LMICs

(MH “Developing Countries”) OR (MH “Africa, Central”) OR (MH “Africa, Northern”) OR (MH “Africa, Western”) OR (MH “Africa, Eastern”) OR (MH “Africa, Southern”) OR Africa OR Afghanistan OR Albania OR Algeria OR Angola OR Antigua OR Barbuda OR Argentina OR Armenia OR Armenian OR Aruba OR Azerbaijan OR Bahrain OR Bangladesh OR Barbados OR Benin OR Byelarus OR Byelorussian OR Belarus OR Belorussian OR Belorussia OR Belize OR Bhutan OR Bolivia OR Bosnia OR Herzegovina OR Hercegovina OR Botswana OR Brazil OR Bulgaria OR “Burkina Faso” OR “Burkina Fasso” OR “Upper Volta” OR Burundi OR Urundi OR Cambodia OR “Khmer Republic” OR Kampuchea OR Cameroon OR Cameroons OR Cameron OR Camerons OR “Cape Verde” OR “Central African Republic” OR Chad OR Chile OR China OR Colombia OR Comoros OR “Comoro Islands” OR Comores OR Mayotte OR Congo OR Zaire OR “Costa Rica” OR “Cote d'Ivoire” OR “Ivory Coast” OR Croatia OR Cuba OR Cyprus OR Czechoslovakia OR “Czech Republic” OR Slovakia OR “Slovak Republic” OR Djibouti OR “French Somaliland” OR Dominica OR “Dominican Republic” OR “East Timor” OR “East Timur” OR “Timor Leste” OR Ecuador OR Egypt OR “United Arab Republic” OR “El Salvador” OR Eritrea OR Estonia OR Ethiopia OR Fiji OR Gabon OR “Gabonese Republic” OR Gambia OR Gaza OR “Georgia Republic” OR “Georgian Republic” OR Ghana OR “Gold Coast” OR Greece OR Grenada OR Guatemala OR Guinea OR Guam OR Guiana OR Guyana OR Haiti OR Honduras OR Hungary OR India OR Maldives OR Indonesia OR Iran OR Iraq OR Jamaica OR Jordan OR Kazakhstan OR Kazakh OR Kenya OR Kiribati OR Korea OR Kosovo OR Kyrgyzstan OR Kirghizia OR Kyrgyz OR Kirghiz OR Kirgizstan OR “Lao PDR” OR Laos OR Latvia OR Lebanon OR Lesotho OR Basutoland OR Liberia OR Libya OR Lithuania OR Macedonia OR Madagascar OR Malagasy OR Malaysia OR Malaya OR Malay OR Sabah OR Sarawak OR Malawi OR Nyasaland OR Mali OR Malta OR “Marshall Islands” OR Mauritania OR Mauritius OR “Agalega Islands” OR Mexico OR Micronesia OR “Middle East” OR Moldova OR Moldovia OR Moldovian OR Mongolia OR Montenegro OR Morocco OR Ifni OR Mozambique OR Myanmar OR Myanma OR Burma OR Namibia OR Nepal OR “Netherlands Antilles” OR “New Caledonia” OR Nicaragua OR Niger OR Nigeria OR “Mariana Islands” OR Oman OR Muscat OR Pakistan OR Palau OR Palestine OR Panama OR Paraguay OR Peru OR Philippines OR Philipines OR Phillipines OR Phillippines OR Poland OR Portugal OR “Puerto Rico” OR Romania OR Rumania OR Roumania OR Russia OR Russian OR Rwanda OR Ruanda OR “Saint Kitts” OR “St Kitts” OR Nevis OR “Saint Lucia” OR “St Lucia” OR “Saint Vincent” OR “St Vincent” OR Grenadines OR Samoa OR “Samoan Islands” OR “Navigator Island” OR “Navigator Islands” OR “Sao Tome” OR “Saudi Arabia” OR Senegal OR Serbia OR Montenegro OR Seychelles OR “Sierra Leone” OR Slovenia OR “Sri Lanka” OR Ceylon OR “Solomon Islands” OR Somalia OR Sudan OR Suriname OR Surinam OR Swaziland OR Syria OR Tajikistan OR Tadzhikistan OR Tadjikistan OR Tadzhik OR Tanzania OR Thailand OR Togo OR Togolese OR Tonga OR Trinidad OR Tobago OR Tunisia OR Turkey OR Turkmenistan OR Turkmen OR Uganda OR Ukraine OR Uruguay OR USSR OR “Soviet Union” OR “Union of Soviet Socialist Republics” OR Uzbekistan OR Uzbek OR Vanuatu OR “New Hebrides” OR Venezuela OR Vietnam OR “Viet Nam” OR “West Bank” OR Yemen OR Yugoslavia OR Zambia OR Zimbabwe OR Rhodesia OR TI “low- and middle- income” OR TI “low income” OR TI “low resource” OR AB “low resource” OR AB “low income” OR AB “low- and middle- income”

### Cochrane Library

#### Concept 1. Mental Health

[mh “Depression”] or [mh “Bipolar disorder”] or [mh “Depressive disorder”] or [mh “mental health”] or [mh “community mental health services”] or [mh “mental health services”] or [mh “psychiatric rehabilitation”] or [mh “psychiatric nursing”] or [mh “mental disorders”] or [mh “dementia”] or [mh “schizophrenia”] or [mh “developmental disabilities”] or “mental health”:ti,ab,kw or “mental healthcare”:ti,ab,kw or “mental illness”:ti,ab,kw or “mental disorder”:ti,ab,kw or “mental disorders”:ti,ab,kw or “disabled”:ti,ab,kw or “disability”:ti,ab,kw or “disabilities”:ti,ab,kw OR “neurologic disorder”:ti,ab,kw or “depression”:ti,ab,kw or “depressive” :ti,ab,kw or “depressed”:ti,ab,kw or “PTSD”:ti,ab,kw or “psychosis”:ti,ab,kw or “psychoses”:ti,ab,kw or “psychotic”:ti,ab,kw or “schizophrenia”:ti,ab,kw or “bipolar”:ti,ab,kw or “epilepsy”:ti,ab,kw or “seizures”:ti,ab,kw or “autism” :ti,ab,kw or “autistic” :ti,ab,kw or “dementia”:ti,ab,kw or “substance abuse”:ti,ab,kw or “drug abuse”:ti,ab,kw or “overuse”:ti,ab,kw or “substance dependence”:ti,ab,kw or “drug dependence”:ti,ab,kw or “harmful use”:ti,ab,kw or “hazardous use”:ti,ab,kw or “suicide”:ti,ab,kw or “self-harm”:ti,ab,kw or “mental retardation”:ti,ab,kw or “neurotic”:ti,ab,kw or “psychotropic” :ti,ab,kw or “anxiolytics”:ti,ab,kw or “depressant”:ti,ab,kw or “epileptic”:ti,ab,kw or “mood stabilizers”:ti,ab,kw or “psychosocial support”:ti,ab,kw or “psychology”:ti,ab,kw or “psychological”:ti,ab,kw or “psychotherapy”:ti,ab,kw or “rehabilitation”:ti,ab,kw or “stigma”:ti,ab,kw or “support group”:ti,ab,kw or “cognitive therapy”:ti,ab,kw OR “reality therapy”:ti,ab,kw OR “behavior therapy”:ti,ab,kw OR “behaviour therapy”:ti,ab,kw or “self-help group”:ti,ab,kw or “alcoholism”:ti,ab,kw or “alcoholic”:ti,ab,kw

#### Concept 2. Community health workers

[mh “community health workers”] or [mh “allied health personnel”] or “health extension worker”:ti,ab,kw or “health extension workers”:ti,ab,kw or “community health worker”:ti,ab,kw or “community health workers”:ti,ab,kw or “community health aide”:ti,ab,kw or “home health aide”:ti,ab,kw or “community health representative”:ti,ab,kw or “community health representatives”:ti,ab,kw or “community networks”:ti,ab,kw or “peer group”:ti,ab,kw or “lay volunteer”:ti,ab,kw or “lay worker”:ti,ab,kw or “lay health worker”:ti,ab,kw or “lay health workers”:ti,ab,kw or “lay health advisor”:ti,ab,kw or “lay health advisors”:ti,ab,kw or “barefoot doctor”:ti,ab,kw or “barefoot doctors”:ti,ab,kw or “peer to peer”:ti,ab,kw or “community based practitioner”:ti,ab,kw or “community based practitioners”:ti,ab,kw or “community-based practitioner”:ti,ab,kw or “community-based practitioners”:ti,ab,kw or “Accredited social health activist”:ti,ab,kw or “Accredited social health activists”:ti,ab,kw or “village health worker”:ti,ab,kw or “village health workers”:ti,ab,kw or “village health guide”:ti,ab,kw or “village health guides”:ti,ab,kw or “village health support guide”:ti,ab,kw or “village health support guides”:ti,ab,kw or “health auxiliary worker”:ti,ab,kw or “health auxiliary workers”:ti,ab,kw or “front-line health worker”:ti,ab,kw or “front-line health workers”:ti,ab,kw or “Shasthyo Sebikas”:ti,ab,kw or “Community Outreach Worker”:ti,ab,kw or “Community Outreach Workers”:ti,ab,kw or “Peer counsellor”:ti,ab,kw or “Peer counsellors”:ti,ab,kw or “Peer counselor”:ti,ab,kw or “Peer counselors”:ti,ab,kw or “Peer Counselour”:ti,ab,kw or “Peer Counselours”:ti,ab,kw or Promotora:ti,ab,kw or “peer educator”:ti,ab,kw or “peer educators”:ti,ab,kw OR “non-physician healthcare worker”:ti,ab,kw OR “non-physician healthcare workers”:ti,ab,kw or “task-shifting”:ti,ab,kw or “task shifting”:ti,ab,kw or “task-sharing”:ti,ab,kw

#### Concept 3. LMICs

[mh “Developing Countries”] OR [mh “Africa, Central”] OR [mh “Africa, Northern”] OR [mh “Africa, Western”] OR [mh “Africa, Eastern”] OR [mh “Africa, Southern”] OR Africa OR Afghanistan OR Albania OR Algeria OR Angola OR Antigua OR Barbuda OR Argentina OR Armenia OR Armenian OR Aruba OR Azerbaijan OR Bahrain OR Bangladesh OR Barbados OR Benin OR Byelarus OR Byelorussian OR Belarus OR Belorussian OR Belorussia OR Belize OR Bhutan OR Bolivia OR Bosnia OR Herzegovina OR Hercegovina OR Botswana OR Brazil OR Bulgaria OR “Burkina Faso” OR “Burkina Fasso” OR “Upper Volta” OR Burundi OR Urundi OR Cambodia OR “Khmer Republic” OR Kampuchea OR Cameroon OR Cameroons OR Cameron OR Camerons OR “Cape Verde” OR “Central African Republic” OR Chad OR Chile OR China OR Colombia OR Comoros OR “Comoro Islands” OR Comores OR Mayotte OR Congo OR Zaire OR “Costa Rica” OR “Cote d'Ivoire” OR “Ivory Coast” OR Croatia OR Cuba OR Cyprus OR Czechoslovakia OR “Czech Republic” OR Slovakia OR “Slovak Republic” OR Djibouti OR “French Somaliland” OR Dominica OR “Dominican Republic” OR “East Timor” OR “East Timur” OR “Timor Leste” OR Ecuador OR Egypt OR “United Arab Republic” OR “El Salvador” OR Eritrea OR Estonia OR Ethiopia OR Fiji OR Gabon OR “Gabonese Republic” OR Gambia OR Gaza OR “Georgia Republic” OR “Georgian Republic” OR Ghana OR “Gold Coast” OR Greece OR Grenada OR Guatemala OR Guinea OR Guam OR Guiana OR Guyana OR Haiti OR Honduras OR Hungary OR India OR Maldives OR Indonesia OR Iran OR Iraq OR Jamaica OR Jordan OR Kazakhstan OR Kazakh OR Kenya OR Kiribati OR Korea OR Kosovo OR Kyrgyzstan OR Kirghizia OR Kyrgyz OR Kirghiz OR Kirgizstan OR “Lao PDR” OR Laos OR Latvia OR Lebanon OR Lesotho OR Basutoland OR Liberia OR Libya OR Lithuania OR Macedonia OR Madagascar OR Malagasy OR Malaysia OR Malaya OR Malay OR Sabah OR Sarawak OR Malawi OR Nyasaland OR Mali OR Malta OR “Marshall Islands” OR Mauritania OR Mauritius OR “Agalega Islands” OR Mexico OR Micronesia OR “Middle East” OR Moldova OR Moldovia OR Moldovian OR Mongolia OR Montenegro OR Morocco OR Ifni OR Mozambique OR Myanmar OR Myanma OR Burma OR Namibia OR Nepal OR “Netherlands Antilles” OR “New Caledonia” OR Nicaragua OR Niger OR Nigeria OR “Mariana Islands” OR Oman OR Muscat OR Pakistan OR Palau OR Palestine OR Panama OR Paraguay OR Peru OR Philippines OR Philipines OR Phillipines OR Phillippines OR Poland OR Portugal OR “Puerto Rico” OR Romania OR Rumania OR Roumania OR Russia OR Russian OR Rwanda OR Ruanda OR “Saint Kitts” OR “St Kitts” OR Nevis OR “Saint Lucia” OR “St Lucia” OR “Saint Vincent” OR “St Vincent” OR Grenadines OR Samoa OR “Samoan Islands” OR “Navigator Island” OR “Navigator Islands” OR “Sao Tome” OR “Saudi Arabia” OR Senegal OR Serbia OR Montenegro OR Seychelles OR “Sierra Leone” OR Slovenia OR “Sri Lanka” OR Ceylon OR “Solomon Islands” OR Somalia OR Sudan OR Suriname OR Surinam OR Swaziland OR Syria OR Tajikistan OR Tadzhikistan OR Tadjikistan OR Tadzhik OR Tanzania OR Thailand OR Togo OR Togolese OR Tonga OR Trinidad OR Tobago OR Tunisia OR Turkey OR Turkmenistan OR Turkmen OR Uganda OR Ukraine OR Uruguay OR USSR OR “Soviet Union” OR “Union of Soviet Socialist Republics” OR Uzbekistan OR Uzbek OR Vanuatu OR “New Hebrides” OR Venezuela OR Vietnam OR “Viet Nam” OR “West Bank” OR Yemen OR Yugoslavia OR Zambia OR Zimbabwe OR Rhodesia OR “low- and middle- income”:ti,ab,kw OR “low income”:ti,ab,kw OR “low resource”:ti,ab,kw

### Pubmed

#### Concept 1. Mental Health

(“Mental Health”[Mesh] or “mental health”[tiab]or “mental healthcare”[tiab] or “Mental Disorders”[Mesh] or “mental illness”[tiab] or “mental disorder”[tiab] or “mental disorders”[tiab] or “disabled”[tiab] or “disability”[tiab] or “disabilities”[tiab] or “Disabled Children”[Mesh] or “Disabled Persons”[Mesh] or “Mentally Disabled Persons”[Mesh] or “neurologic disorder”[tiab] or “Depression”[Mesh] or “depression”[tiab] or “Depressive Disorder”[Mesh] or “depressive”[tiab] or “depressed”[tiab] or “Stress Disorder, Post-Traumatic”[Mesh] or “PTSD”[tiab] or “Psychotic Disorder”[Mesh] or “psychosis”[tiab] or “psychoses”[tiab] or “psychotic”[tiab] or “Schizophrenia”[Mesh] or “schizophrenia”[tiab] or “Bipolar Disorder”[Mesh] or “bipolar”[tiab] or “Epilepsy”[Mesh] or “epilepsy”[tiab] or “Seizures”[Mesh] or “seizures”[tiab] or “Developmental Disabilities”[Mesh] or “Learning Disorders”[Mesh] or “Intellectual Disability”[Mesh] or “Autistic disorder” [Mesh] or “autism” [tiab] or “autistic” [tiab] or “Dementia”[Mesh] or “dementia”[tiab] or “Substance-Related Disorders”[Mesh] or “Substance Abuse, Intravenous”[Mesh] or “Marijuana Abuse”[Mesh] or “Cocaine-Related Disorders”[Mesh] or “Amphetamine-Related Disorders”[Mesh] or “substance abuse”[tiab] or “drug abuse”[tiab] or “overuse”[tiab] or “substance dependence”[tiab] or “drug dependence”[tiab] or “harmful use”[tiab] or “hazardous use”[tiab] or “Suicide”[Mesh] or “suicide”[tiab] or “self-harm”[tiab] or “mental retardation”[tiab] or “neurotic”[tiab] or “Alcoholism”[Mesh] or “Adjustment Disorders”[Mesh] OR “Affective Disorders, Psychotic”[Mesh] or “psychotropic” [tiab] or “anxiolytics”[tiab] or “depressant”[tiab] or “epileptic”[tiab] or “mood stabilizers”[tiab] or “psychosocial support”[tiab] or “psychology”[tiab] or “psychological”[tiab] or “psychotherapy”[tiab] or “rehabilitation”[tiab] or “stigma”[tiab] or “support group”[tiab] or “cognitive therapy”[tiab] OR “reality therapy”[tiab] OR “behavior therapy”[tiab] OR “behaviour therapy”[tiab] or “self-help group”[tiab] or “Self-Help Groups”[Mesh] or “Psychology”[Mesh] or “Psychotherapy”[Mesh] or “Counseling”[Mesh] or “Rehabilitation”[Mesh] or “Social Stigma”[Mesh] or “Resilience, Psychological”[Mesh] or “Discrimination (Psychology)”[Mesh]).

#### Concept 2. Community health workers

(“health extension worker”[tiab] or “health extension workers”[tiab] or “Community Health Workers”[Mesh] or “community health worker”[tiab] or “community health workers”[tiab] or “community health aide”[tiab] or “home health aide”[tiab] or “community health representative”[tiab] or “community health representatives”[tiab] or “community networks”[tiab] or “peer group”[tiab] or “lay volunteer”[tiab] or “lay worker”[tiab] or “lay health worker”[tiab] or “lay health workers”[tiab] or “lay health advisor”[tiab] or “lay health advisors”[tiab] or “barefoot doctor”[tiab] or “barefoot doctors”[tiab] or “peer to peer”[tiab] or “community based practitioner”[tiab] or “community based practitioners”[tiab] or “community-based practitioner”[tiab] or “community-based practitioners”[tiab] or “Accredited social health activist”[tiab] or “Accredited social health activists”[tiab] or “village health worker”[tiab] or “village health workers”[tiab] or “village health guide”[tiab] or “village health guides”[tiab] or “village health support guide”[tiab] or “village health support guides”[tiab] or “health auxiliary worker”[tiab] or “health auxiliary workers”[tiab] or “front-line health worker”[tiab] or “front-line health workers”[tiab] or “Shasthyo Sebikas”[tiab] or “Community Outreach Worker”[tiab] or “Community Outreach Workers”[tiab] or “Peer counsellor”[tiab] or “Peer counsellors”[tiab] or “Peer counselor”[tiab] or “Peer counselors”[tiab] or “Peer Counselour”[tiab] or “Peer Counselours”[tiab] or Promotora[tiab] or “peer educator”[tiab] or “peer educators”[tiab] OR “non-physician healthcare worker”[tiab] OR “non-physician healthcare workers”[tiab] or “task-shifting”[tiab] or “task shifting”[tiab] or “task-sharing”[tiab] or “lay counselor”[tiab] or “lay counselors”[tiab])

#### Concept 3. LMICs

(Africa[tw] OR Afghanistan [tw] OR Albania [tw] OR Algeria [tw] OR Angola [tw] OR Antigua [tw] OR Barbuda [tw] OR Argentina [tw] OR Armenia [tw] OR Armenian [tw] OR Aruba [tw] OR Azerbaijan [tw] OR Bahrain [tw] OR Bangladesh [tw] OR Barbados [tw] OR Benin [tw] OR Byelarus [tw] OR Byelorussian [tw] OR Belarus [tw] OR Belorussian [tw] OR Belorussia [tw] OR Belize [tw] OR Bhutan [tw] OR Bolivia [tw] OR Bosnia [tw] OR Herzegovina [tw] OR Hercegovina [tw] OR Botswana [tw] OR Brazil [tw] OR Bulgaria [tw] OR “Burkina Faso” [tw] OR “Burkina Fasso” [tw] OR “Upper Volta” [tw] OR Burundi [tw] OR Urundi [tw] OR Cambodia [tw] OR “Khmer Republic” [tw] OR Kampuchea [tw] OR Cameroon [tw] OR Cameroons [tw] OR Cameron [tw] OR Camerons [tw] OR “Cape Verde” [tw] OR “Central African Republic” [tw] OR Chad [tw] OR Chile [tw] OR China [tw] OR Colombia [tw] OR Comoros [tw] OR “Comoro Islands” [tw] OR Comores [tw] OR Mayotte [tw] OR Congo [tw] OR Zaire [tw] OR “Costa Rica” [tw] OR “Cote d'Ivoire” [tw] OR “Ivory Coast” [tw] OR Croatia [tw] OR Cuba [tw] OR Cyprus [tw] OR Czechoslovakia [tw] OR “Czech Republic” [tw] OR Slovakia [tw] OR “Slovak Republic” [tw] OR Djibouti [tw] OR “French Somaliland” [tw] OR Dominica [tw] OR “Dominican Republic” [tw] OR “East Timor” [tw] OR “East Timur” [tw] OR “Timor Leste” [tw] OR Ecuador [tw] OR Egypt [tw] OR “United Arab Republic” [tw] OR “El Salvador” [tw] OR Eritrea [tw] OR Estonia [tw] OR Ethiopia [tw] OR Fiji [tw] OR Gabon [tw] OR “Gabonese Republic” [tw] OR Gambia [tw] OR Gaza [tw] OR “Georgia Republic” [tw] OR “Georgian Republic” [tw] OR Ghana [tw] OR “Gold Coast” [tw] OR Greece [tw] OR Grenada [tw] OR Guatemala [tw] OR Guinea [tw] OR Guam [tw] OR Guiana [tw] OR Guyana [tw] OR Haiti [tw] OR Honduras [tw] OR Hungary [tw] OR India [tw] OR Maldives [tw] OR Indonesia [tw] OR Iran [tw] OR Iraq [tw] OR Jamaica [tw] OR Jordan [tw] OR Kazakhstan [tw] OR Kazakh [tw] OR Kenya [tw] OR Kiribati [tw] OR Korea [tw] OR Kosovo [tw] OR Kyrgyzstan [tw] OR Kirghizia [tw] OR Kyrgyz [tw] OR Kirghiz [tw] OR Kirgizstan [tw] OR “Lao PDR” [tw] OR Laos [tw] OR Latvia [tw] OR Lebanon [tw] OR Lesotho [tw] OR Basutoland [tw] OR Liberia [tw] OR Libya [tw] OR Lithuania [tw] OR Macedonia [tw] OR Madagascar [tw] OR Malagasy [tw] OR Malaysia [tw] OR Malaya [tw] OR Malay [tw] OR Sabah [tw] OR Sarawak [tw] OR Malawi [tw] OR Nyasaland [tw] OR Mali [tw] OR Malta [tw] OR “Marshall Islands” [tw] OR Mauritania [tw] OR Mauritius [tw] OR “Agalega Islands” [tw] OR Mexico [tw] OR Micronesia [tw] OR “Middle East” [tw] OR Moldova [tw] OR Moldovia [tw] OR Moldovian [tw] OR Mongolia [tw] OR Montenegro [tw] OR Morocco [tw] OR Ifni [tw] OR Mozambique [tw] OR Myanmar [tw] OR Myanma [tw] OR Burma [tw] OR Namibia [tw] OR Nepal [tw] OR “Netherlands Antilles” [tw] OR “New Caledonia” [tw] OR Nicaragua [tw] OR Niger [tw] OR Nigeria [tw] OR “Mariana Islands” [tw] OR Oman [tw] OR Muscat [tw] OR Pakistan [tw] OR Palau [tw] OR Palestine [tw] OR Panama [tw] OR Paraguay [tw] OR Peru [tw] OR Philippines [tw] OR Philipines [tw] OR Phillipines [tw] OR Phillippines [tw] OR Poland [tw] OR Portugal [tw] OR “Puerto Rico” [tw] OR Romania [tw] OR Rumania [tw] OR Roumania [tw] OR Russia [tw] OR Russian [tw] OR Rwanda [tw] OR Ruanda [tw] OR “Saint Kitts” [tw] OR “St Kitts” [tw] OR Nevis [tw] OR “Saint Lucia” [tw] OR “St Lucia” [tw] OR “Saint Vincent” [tw] OR “St Vincent” [tw] OR Grenadines [tw] OR Samoa [tw] OR “Samoan Islands” [tw] OR “Navigator Island” [tw] OR “Navigator Islands” [tw] OR “Sao Tome” [tw] OR “Saudi Arabia” [tw] OR Senegal [tw] OR Serbia [tw] OR Montenegro [tw] OR Seychelles [tw] OR “Sierra Leone” [tw] OR Slovenia [tw] OR “Sri Lanka” [tw] OR Ceylon [tw] OR “Solomon Islands” [tw] OR Somalia [tw] OR Sudan [tw] OR Suriname [tw] OR Surinam [tw] OR Swaziland [tw] OR Syria [tw] OR Tajikistan [tw] OR Tadzhikistan [tw] OR Tadjikistan [tw] OR Tadzhik [tw] OR Tanzania [tw] OR Thailand [tw] OR Togo [tw] OR Togolese [tw] OR Tonga [tw] OR Trinidad [tw] OR Tobago [tw] OR Tunisia [tw] OR Turkey [tw] OR Turkmenistan [tw] OR Turkmen [tw] OR Uganda [tw] OR Ukraine [tw] OR Uruguay [tw] OR USSR [tw] OR “Soviet Union” [tw] OR “Union of Soviet Socialist Republics” [tw] OR Uzbekistan [tw] OR Uzbek [tw] OR Vanuatu [tw] OR “New Hebrides” [tw] OR Venezuela [tw] OR Vietnam OR “Viet Nam” [tw] OR “West Bank” [tw] OR Yemen [tw] OR Yugoslavia [tw] OR Zambia [tw] OR Zimbabwe [tw] OR Rhodesia [tw] OR ((developing [TiAB] OR “less developed” [TiAB] OR “under developed” [TiAB] OR underdeveloped [TiAB] OR “middle income” [TiAB] OR “low income” [TiAB] OR “lower income” [TiAB] OR underserved [TiAB] OR “under served” [TiAB] OR deprived [TiAB] OR poor* [TiAB]) AND (countr* [TiAB] OR nation* [TiAB] OR population* [TiAB] OR world [TiAB])) OR ((transitional [TiAB] OR developing [TiAB] OR “less developed” [TiAB] OR “lesser developed” [TiAB] OR “under developed” [TiAB] OR underdeveloped [TiAB] OR middle income [TiAB] OR “lower income” [TiAB] OR “lower income” [TiAB]) AND (economy [TiAB] OR economies [TiAB])) OR “low resource”[tiab] OR “low-resource”[tiab])
